# Supplementary material for: Are carbon emissions trading and green financial instruments synergistic? -Comprehensive quantitative research based on content analysis
Source: PLoS One. 2024 Mar 7;19(3):e0298601. doi: 10.1371/journal.pone.0298601 (PMC10919601; doi:10.1371/journal.pone.0298601)
Supplement: S1 Data — (ZIP) [file pone.0298601.s001.zip › Data/地级市Z_F工作报告.zip!/║■▒▒/╦μ╓▌╩╨/└┤╫╘╣1⁄2╓┌║┼í╛┬φ┐╦╩2╛▌═°í┐_2015.docx]

各位代表：

现在，我代表市人民政府向大会报告政府工作，请予审议，并请各位政协委员和列席人员提出意见。

一、2014年工作回顾

刚刚过去的2014年，面对复杂严峻形势，我们在省委、省政府和市委的坚强领导下，紧紧依靠全市人民，坚持稳中求进、改革创新、特色突破，较好完成了市三届人大四次会议确定的各项工作任务，在推动“圣地车都”建设的征程中迈出坚实步伐。

—— 经济实力持续增强。全面完成省定目标任务，各项指标增速好于优于全国、全省平均水平，主要经济指标增速实现“保五争三”。预计完成地区生产总值725亿 元，同比增长9.5%。全社会固定资产投资768.8亿元，增长22.3%，增速居全省第二位；规模工业增加值366亿元，增长11%，增速并列全省第四 位；地方公共财政预算收入36.7亿元，增长23.6%，增速居全省第三位；外贸出口12.7亿美元，增长13.1%；社会消费品零售总额354.4亿 元，增长13.6%，增速居全省第三位。

—— 发展质效持续提升。产业结构不断优化，二三产业占比进一步提高。关键指标全面上行，工业用电量增长8.81%，增速居全省第二位；营业性道路货运量增长 15.1%；新增贷款68亿元，贷存比提高2个百分点。工业品产销率99.1%，居全省第一位。创新驱动成效显著，新增16家高新技术企业、5个中国驰名 商标；实现战略性新兴产业增加值44亿元，增长9.5%；高新技术产业增加值66.5亿元，增长13%，增速高于规模工业2.2个百分点。财政收入质量进 一步提升，税收占公共预算收入的比重继续提高。

—— 项目驱动持续发力。谋划实施5个投资50亿元左右的大项目，开启随州“大项目时代”。其中地面光伏电站、区域电网改造和500千伏变电站启动建设，鄂北水 资源配置工程和武汉至西安客运专线随州段即将开工，华能随州2×66万千瓦燃煤电厂项目获国家批准。项目投资、对上争资都创历史新高，在建亿元以上项目 220个、完成投资330亿元，对上争取无偿资金49.8亿元。麻竹高速随州西段全线贯通，我市高速公路万人拥有量全省第一，白云大道延伸和编钟大道浪河 段建成通车，滨湖体育运动场、新政务服务中心及城市规划展览馆投入使用，宁西铁路增建二线、东外环公路、传媒中心建设按期推进，高新区新建鹿鹤大道等8条 园区道路。东风车轮新工厂、航天双龙新能源汽车等一批重点工业项目启动实施。华润凤鸣风电场投入运行，马鞍山100兆瓦光伏电站和界山、中华山、花山风电 场建设全面推进，随州成为全省新能源第一大市。

—— 人民生活持续改善。财政用于民生领域的支出达到90.8亿元，占公共财政预算支出的81.3%，占比高于全省6.3个百分点。全市城镇、农村居民人均可支 配收入分别增长9.1%和12%，农村居民收入增幅连续三年高于城镇居民。市政府年初承诺的扶持创业带动就业、安居工程建设、扶贫开发等“十件实事”全面 完成。

一年来，我们突出“五抓”，许多工作实现新突破。

（一）抓改革开放，发展活力明显增强。

在行政审批制度改革上，全面清理审批事项，市本级取消、下放55项，再造审批流程，推行并联审批、代办服务、建设项目“一表制”收费。实行注册资本认缴 制、企业年报公示制、先照后证等措施，有效激发了市场活力。在财政金融改革上，进一步完善部门预算、政府采购等管理机制，出台财政项目资金绩效评价实施办 法，构建了国资监管制度体系。资本市场加快发展，湖北银行落户开业，裕国菇业登陆新三板，4家企业挂牌新四板，成功发行10亿元建投债。推出“助保贷”、 “政银集合贷”，为中小微企业发放贷款5.74亿元。市和各县市区继续获评“金融信用市县”。在农村产权制度改革上，随县在全省率先完成土地确权登记颁证 工作，广水市、曾都区按计划顺利推进，全市新增流转土地8.4万亩、总面积近50万亩，环潭镇、洪山镇农村综合产权交易试点取得成功。在机构体制改革上， 市县两级卫生计生机构改革全面完成，公立医院改革加快推进，疾病应急救助制度、基本药物制度不断完善，大洪山景区医疗、养老“两室联建、医养融合”试点效 果良好。市、县、乡镇三级食品药品监管体制全面建立。在扩大对外开放上，外贸出口总量稳步攀升、结构不断优化，香菇出口全国第一，茶叶出口全省第一，机电 产品出口全省第三，成功创建省出口专用车产品质量安全示范区。办理出口退免税10亿元，增幅全省第一。招商引资量质齐升，落户央企省企累计达到19家，新 签约项目120个，其中工业项目90个。全市实际利用内联资金196亿元，利用外资近1亿美元，分别增长25.5%和13.5%。

（二）抓结构优化，转型升级凸显亮点。

现代农业稳步发展。粮食总产165.5万吨，增产7.3万吨，创历史最好水平。整合涉农资金11.5亿元，支持农业产业化发展，食用菌、马铃薯、畜禽等特 色种养业稳步壮大。新增农民合作社398家，获评国家示范社10家；新登记家庭农场57家，获评省示范农场9家。

加快农村基础设施建设，完成72座小（二）型水库除险加固，解决了16万农村人口安全饮水问题。争取高产农田整理项目10个，面积12万亩。建成通村公路 379公里，二级以上公路通达所有乡镇。工业经济企稳回升。多措并举帮扶企业应对经济下行，在困难比预想多的情况下，取得比预期好的结果。全年实现规模工 业总产值1220亿元，增长11%。“六大支柱产业”支撑有力，拉动工业增长14.5个百分点。汽车机械、食品工业产值双双突破350亿元，铸造产业成为 我市第4个省重点产业集群。以央视曝光为契机，闻声而动，下重手、严管理，深入推进专汽行业整顿、资源整合，严厉打击非法产销行为，化危为机，变被动为主 动，专汽产品质量稳步提高，市场秩序显著好转，我市被授予国家首批专汽产业区域品牌建设试点、国家专用汽车高新技术产业化基地、湖北省新型工业化产业示范 基地，“中国专汽之都”品牌形象进一步巩固提升。“六个五十”重点项目建设成效明显，50个亿元以上项目和50个3000万元以上技改项目完成投资80亿 元。中小企业加快成长，新增规模企业86家，亿元以上企业突破400家。我市入围湖北民企百强企业达到9家，位列“一主两副”城市之后，居全省第四位。第 三产业升温提速。甲午年寻根节成功举办，文化旅游快速发展。大洪山保护开发纳入省级统筹，获批全省生态旅游示范区，核心景区开放迎宾，文化公园全面建成， 千年银杏谷“黄金月”火爆有序，3个景区同步跻身国家4A级景区。炎帝故里、博物馆、曾侯乙墓联合创建5A级景区通过国家景观质量评审。全市接待游客 1583.5万人次，旅游综合收入97.1亿元，分别增长9.2%和7.3%。现代服务业逐步繁荣，电子商务加快发展，“淘宝网·特色中国—随州馆”获批 开馆，首届青岛啤酒节效益良好。农资、卷烟、成品油等专业市场监管得到加强，居民消费价格涨幅低于全省平均水平。县域经济实力增强。曾都区继续保持全省第 一方阵，广水市、随县综合竞争力继续提升。高新区生态产业新城建设有序展开，创建国家高新区通过科技部初审。

（三）抓城乡统筹，神韵随州再展新颜。“3+3”城市品牌创建圆满收官，我市被正式授予国家园林城市、国家森林城市和省级环保模范城市，顺利通过国家历史 文化名城和省级文明城市、卫生城市复查，全市城镇化率提高2个百分点。城乡规划更加科学。继续优化城市空间布局，编制完成全市城镇化发展战略、综合交通体 系、城南片区城市设计等规划295项。城市功能更加完善。主城区实施49项重点市政项目，青年路“两路一桥”全线贯通，建成一批压缩式垃圾中转站，主城区 背街小巷全部硬化亮化，新增绿地21万平方米，东护城河治理、美食文化街、随州农副产品物流园等一批项目相继开工。随县同步推进新城拓展、旧城改造、开发 区和炎帝故里景区4个主功能区建设；广水市“一河两岸”初具规模，跻身省级文明城市。城市管理更加有序。推行相对集中行政处罚权管理模式，基本建成数字化 城管指挥中心。启用智能交通综合管控系统，创新摩托车、电动车管理体制机制，集中整治“马路市场”，小区物业管理专项整治试点初见成效。乡村面貌更加靓 丽。深化“洁美家园大行动”，加强农村生活垃圾治理，我市在全省首次考评中排名第三，曾都区被纳入全省整县推进试点。高新区美丽乡村示范片建设扎实推进， 全市7个乡镇入选全国重点镇，3个村入选第三批中国传统村落保护名录，10个村成为省宜居村庄，8个村获评省绿色示范乡村，大洪山长岗镇、广水桃源村分别 荣登全省十大最美乡镇、乡村榜首。

（四）抓民生改善，社会大局和谐稳定。积极做好就业和社会保障工作。增发创业就业小额担保贷款1.3亿元，城镇新增就业2.3万人，创业带动就业2950 人。五项社会保险扩面近1万人，城乡居民最低生活保障、企业退休人员和被征地农民养老保险待遇继续提标；实施疾病应急救助制度，完善重大疾病保障制度，医 疗服务水平进一步提高。倾力关爱弱势群体，发放各类救助资金1.2亿元，免费配发残疾人辅助器具2060件。探索推行社会化养老模式，新建城乡社区养老服 务中心239个。加快安居工程建设，保障性住房和棚户区改造住房新开工8744套，分配入住3823套，新增租赁补贴708户。住房公积金扩面8550 人，新增归集4.5亿元，当年使用4.1亿元、惠及5500户家庭。协调发展社会事业。优先发展教育事业，新建和改扩建幼儿园42所、农村初中校舍3.5 万平方米，发放贫困生资助金4600多万元，我市成为全省首批义务教育均衡发展全覆盖城市；高考上线人数大幅增加，职业教育基础能力继续提升。卫生计生事 业取得成效，规范化建设459个村卫生室，医德医风和医疗质量不断改进，免费为9万多名农村妇女进行了“两癌”筛查；强化出生人口性别比源头治理，单独两 孩政策全面实施。文化体育事业繁荣发展，博物馆二期建设如期完工，创建国家大遗址保护片区取得进展，建成133处体育健身工程，随县、广水市被授予省民间 文化艺术之乡，安居镇获评中国历史文化名镇。广电影视、通信事业加快发展，广播电视村村通工程实现全覆盖，新兴媒体的影响力日益增强。进一步创新社会治 理。围绕建设法治随州，多个层面的法治创建活动有效开展，在全省率先落实行政首长出庭应诉制，法律援助案件质量位居全省前列。继续推进“三民工程”，累计 为居民代办事项16万件。加强城乡网格化管理，着力构建矛盾纠纷大调解格局，化解信访积案1100件。加快完善治安防控体系，坚持“六警合一”巡防机制， 连续两年实现命案全破，成功侦破特大走私武器弹药和盗掘古墓、集资诈骗等重大案件，缉毒管控等多项工作进入全省第一方阵，群众安全感、平安创建知晓率和参 与度等指标位居全省前列。严格落实安全生产责任制，公共安全形势持续稳定。加大环境保护监管力度，开展执法“零点行动”，打响城区饮用水源地治理保护攻坚 战，“五到位”取得阶段性成果。大力弘扬社会主义核心价值观，加强思想道德建设，王何林等11名道德模范入选中国好人榜、湖北好人榜，掀起“讲文明树新 风”活动热潮，社会文明程度不断提升。

（五）抓政府自身建设，干部作风不断改进。依法履行政府职能，自觉接受人大及其常委会的法律监督和政协的民主监督，认真听取民主党派、工商联和社会各界的 意见建议。办理建议提案225件，满意率和基本满意率达到98%以上。扎实开展党的群众路线教育实践活动，认真贯彻落实中央八项规定和省委六条意见，狠刹 “四风”，坚决“除陋习、刹歪风、树正气”。以赶情风专项整治、民生保障投入提升等“八大工程”为抓手，发挥电视问政、周报月评年考、乡镇大比武等机制的 倒逼效应，解决了一批企业和群众反映强烈的突出问题。加大明察暗访力度，查处了一批典型案件，经济发展环境不断优化。全市查处违反八项规定问题303个、 处理406人，“三公”经费下降37.6%，降幅居全省前列。加强党风廉政建设，狠抓各级政府及其部门党委（党组）主体责任落实，反腐倡廉民意调查满意度 继续保持全省领先。

新闻出版、文联、社科联、外事侨务、对台、民族宗教、移民、对口援疆、国防教育动员和双拥工作得到新的加强，工会、青年、妇女儿童、红十字会、无偿献血、 老龄和慈善事业有了新的发展，审计、统计、国调、机关事务、档案、保密和地方志工作取得新的进步，烟草、邮政、无线电、人防、消防、水文、气象等工作都收 获新的成果！

各位代表！成绩来之不易，这是省委、省政府正确领导、亲切关怀的结果，是市委统揽全局、科学决策的结果，是市人大、市政协加强监督、参政议政的结果，是全 市人民和衷共济、克难奋进的结果！在此，我代表市人民政府，向全市人民，向所有纳税人，向各位人大代表、政协委员，向各民主党派、工商联、人民团体和无党 派人士，向驻随部队、武警官兵和政法公安干警，向所有关心支持随州发展的老领导和各界朋友，表示衷心的感谢并致以崇高的敬意！

在总结成绩的同时，我们也清醒看到，前进道路上还面临不少困难和问题：一是经济增长基础不牢。受宏观经济下行影响，固定资产投资、工业增加值等部分经济指 标增速不同程度回落，一些重点企业生产下滑。二是要素保障明显不足。国家环保、土地、林地政策趋紧，融资难、融资贵和用工结构性矛盾更加显现，部分企业生 产经营成本上升。三是公共服务保障不够。市民反映强烈的交通拥堵、环境质量不优、教育质量不高等问题需要进一步解决，依法治理、安全生产、食品安全、校园 安全等基层基础工作还较薄弱。四是少数部门和工作人员效能不高。表现在对新常态认识不深、把握不准、适应能力不够，有的工作责任心和服务意识不强，为官不 为，政务环境有待进一步优化，作风建设还需向深度用力。对这些问题，我们一定高度重视，综合施策，着力加以解决。

二、2015年形势和任务

2015年是全面深化改革的关键之年，是全面推进依法治国的开局之年，是完成“十二五”规划的收官之年，也是加快“圣地车都”建设的承前启后之年。新常态 背景下，虽然我们面临一系列风险挑战，但仍然拥有多重叠加的重大机遇。一是宏观环境趋稳向好。尽管世界经济仍处于深度调整期，但总体缓慢回升，为我们用好 两个市场、两种资源，进一步扩大对外开放，特别是专汽、电子、农产品等特色产品走出去拓展了空间。二是新常态蕴含重大契机。新常态凸显“中高速、优结构、 新动力、多挑战”四大鲜明特征，显露出我国经济稳进的“姿态”、累积的“富态”、结构的“优态”和市场的“活态”，表明尽管经济增速有所放缓，但实际增量 依然可观，增长动力更为多元，发展前景更加稳定，市场活力进一步释放。特别是呈现的消费需求、投资需求、市场竞争等“九大趋势性变化”，更为我们发现和培 育新的增长点指明了方向路径。三是政策利好因素较多。中央继续实施积极的财政政策和稳健的货币政策，微刺激政策定向发力。长江经济带建设上升为国家战略， 湖北中部崛起和“两圈两带”战略纵深推进，必将带动全省整体步入发展快车道。加之我市处于“两圈”节点，被纳入大别山试验区、汉江生态经济带，这些国家、 省、部战略层面的支持，都带来一系列重大机遇。四是随州发展潜力巨大、蓄势待发。建市以来特别是“十二五”以来，全市经济持续快速增长，呈现跨越式发展良 好势头。随着“大项目时代”的到来，大投入、大建设、大产出的态势强劲有力，发展的底盘更加稳固。我市草根经济坚韧顽强，全民创业热情持续高涨，去年新登 记各类市场主体突破3万户，再创历史新高。汽车机械、农产品加工、新能源、文化旅游等产业形成特色，城市的品牌、魅力和影响力不断彰显。这些都为我们加快 转型跨越提供了有利条件，只要我们科学把握形势，保持足够的战略定力，遵循规律，趋利避害，奋发进取，精准施策，就一定能够迎接新挑战、抢抓新机遇、实现 新作为。

今年政府工作的总体要求是：全面贯彻落实党的十八大、十八届三中、四中全会和中央、全省经济工作会议精神，坚持走社会主义法治道路，坚持以全面深化改革统 揽全局，坚持 “市场决定取舍、绿色决定生死、民生决定目的”三维纲要，坚持竞进提质、升级增效、先行先试、特色发展不动摇，着力激发创新活力，着力推动转型升级，着力 强化项目拉动，着力扩大对外开放，着力推进新型城镇化，着力增进民生福祉，全面完成“十二五”规划任务，主动适应新常态，积极争创新业绩，奋力开创“圣地 车都”建设新局面。

今年经济社会发展的主要预期目标是：地区生产总值增长9.2%，规模工业增加值增长10.5%左右，全社会固定资产投资增长20%，地方公共财政预算收入 增长13.5%，外贸出口增长10%，实际利用内联资金、利用外资分别增长20%和10%，社会消费品零售总额增长13%左右，城镇、农村居民人均可支配 收入分别增长9.2%和11%，居民消费价格涨幅控制在3.5%以内，主要污染物总量减排、环境质量控制等全面完成省考核目标。

上述目标是着眼经济发展的新常态和随州转型跨越的新要求，综合分析研究提出来的，是必须确保完成的“底线”。实际工作中，我们将继续实行“三道目标线”管理，保持“竞进”的状态，发扬“跳起来摘桃子”的精神，坚定信心，自我加压，努力实现“保五争三”的赶超目标。

三、2015年重点工作

围绕今年目标任务，必须以提高经济发展质量和效益为中心，重点做好以下工作。

（一）以更强支撑谱写转型跨越新蓝图。强化全域视野和战略思维，科学规划今后五年的发展。坚定不移扩投资，持续用力上项目，力争今年固定资产投资突破1000亿元。

坚持应急与谋远结合，科学编制规划。准确把握经济社会发展总体趋势，充分衔接全面建成小康社会各项目标，立足当前，着眼长远，因地制宜，科学编制“十三 五”规划。突出抓好“十三五”项目库建设，力争入库项目总规模达到1.5万亿元；加强策划包装、沟通对接，力争有更多的重大项目进入国家和省规划安排。出 台我市参与汉江生态经济带、新型城镇化建设实施方案，修订完善重点产业发展、商业网点布局等规划。注重提高县乡规划的可操作性，探索推进县市“多规合 一”，一张蓝图干到底。

坚持建设与储备并重，扩大项目投资。开展项目建设竞进年、项目储备提质年、社会投资促进年三个“主题年”活动。全力抓好在建重大项目，确保麻竹高速浪河互 通及大洪山、长岗连接线建成通车，力争东风车轮新工厂和航天双龙新能源汽车等项目竣工投产，加快生态产业新城“一河两岸”综合整治和神农大道建设，推进和 促成鄂北水资源配置工程、武西客运专线、华能随州燃煤电厂、青岛啤酒搬迁扩能、编钟大道延伸至何店段等项目尽早开工。在抓好续建和新开工项目的同时，及早 谋划下一年的项目储备，确保接续推进。充分调动社会投资积极性，继续推出一批吸引社会资本参与的项目，积极推广政府与社会资本合作（PPP）模式，促进投 资便利化。健全领导包保、“一项一策”、现场观摩、督办通报等工作机制，加强要素协调保障，完善“一表制”收费制度，以最大的力度确保项目建设取得实效。

坚持数量与质量兼顾，抓好招商引资。把招商引资作为扩投资、稳增长的有效支撑，强化目标考核，抓好专班招商、驻点招商、网络招商、委托招商和以商引商，围 绕产业链完善配套，积极引进更多的战略投资者。更加注重精选招商项目，切实提高招商引资质效，力争引进亿元以上项目40个以上，实际利用内联资金235亿 元以上，利用外资1.1亿美元以上。积极抓好争资立项，争取无偿资金增长10%以上。

（二）以更优结构增强特色经济新优势。注重运用市场的力量，培育壮大特色产业，促进新常态下经济发展提质增效。

立足集群发展，壮大支柱产业，做大做强特色工业。以建设“中国专汽之都”、“中国风机名城”和争创国家新能源示范城市为重点，狠抓七项关键举措，加快做大 工业总量，调优工业结构。狠抓传统产业升级，推动汽车机械、农产品加工、医药化工、冶金建材等产业转型发展。突出抓好专汽行业集中整顿，促进行业自律，继 续推行“五统一”管理模式，支持曾都区湖北专用汽车产业园建设，办好2015中国（随州）专汽产业发展国际论坛，争创国家级新型工业化产业示范基地。狠抓 新型产业壮大，扶持发展电子信息、战略性新兴产业，力争界山等3个风电场并网发电，推动马鞍山光伏电站竣工投产，力争全市光伏发电装机容量达到200兆 瓦。狠抓混合所有制经济，深化与央企省企对接合作，积极推进湖北三环与全力公司、中节能与金峰肥业等企业重组，承接健民集团项目向随州转移，力争新增混合 所有制企业5家以上、投资20亿元以上。狠抓县域工业发展，加快构建以随县农产品加工、广水风机、曾都铸造和专用汽车等为支柱的县域工业体系，促进县域经 济特色化、集群化发展。支持高新区“四园两区”建设，做大做优发展平台，培育高新技术产业，确保创建国家高新区一举成功。狠抓创新驱动战略，组建专汽产业 技术创新联盟，加强科技孵化器、专汽研究院创新能力建设，促进科技成果转化，力争高新技术产业增加值占GDP的比重突破10%，新增中国驰名和省著名商标 6件以上、湖北名牌产品5个以上。狠抓市场主体培育，实施重点企业培育、中小企业成长行动方案，力争新增亿元以上企业20家、规模企业60家，培育小微企 业1600户，新登记市场主体2万户以上。狠抓企业家队伍建设，坚持“产业第一、企业家老大”的服务理念，培育重商文化，开办“随州企业家市场大学”，全 面提升企业家思想境界和经营能力。

立足农民增收，建好特色板块，做精做深优质农业。以壮大粮食、食用菌、蔬菜、养殖、林果、农产品加工等特色板块为重点，积极培育新型农业经营主体，加快政 策、资本、项目、科技融合，推进农业现代化进程。大力优化农业产出结构，以发展绿色、特色、品牌农产品为主攻方向，坚持稳粮增收、提高综合效益，推广节水 作物和节水模式，确保粮食增产2.5万吨。进一步扩大畜禽、食用菌、木本油料、蔬菜、应时鲜果等特色基地规模。大力发展农业产业化，加快建立新型农业经营 体系和农产品流通体系，引导农村土地流转，发展多种形式的适度规模经营，提高农业生产集约化程度。鼓励发展家庭农场、农民合作社，推动农产品电商、物流、 冷链等对接直销。突破性发展农产品精深加工，重点扶持农业产业化龙头企业，力争新增省级以上龙头企业2家。加强低丘岗地资源开发利用，探索循环农业、观光 农业发展新模式，多途径增加农民收入。加强农产品质量监管，推进“餐桌污染”治理，保障人民群众“舌尖上的安全”。大力推进农村基础设施建设，高标准改造 基本农田10万亩，建成通村公路350公里。实施中小河流治理、水生态修复等重点水利工程，力争全市水库“全脱险”。

立足拉动消费，加强旅游营销，做活做优现代服务业。以办好乙未年寻根节和第25届中国（随州）兰花博览会为契机，以争创湖北省文化旅游产业发展试验区和鄂 北交通物流中心为重点，加快发展文化旅游等第三产业，力争服务业增加值增长10%以上。大力推进炎帝故里文化旅游区创建5A级景区，全面提升软硬件设施， 力争年内通过验收。启动大洪山5A级景区创建工作，支持抱朴谷隐修主题公园、昱辰观光农业示范园、桃源村、三潭等景区创4A。整合旅游促销资金、平台和渠 道，开发利用新媒体，探索发展智慧旅游，创作一批影视宣传作品，全方位推介炎帝故里、编钟之乡、佛教名山、千年银杏谷、西游神话世界等文化旅游品牌。实施 游客满意度提升工程，力争全年接待游客1700万人次，旅游综合收入达到105亿元。优先发展生产性服务业，推进电子商务区域总部和营运中心、大宗农产品 交易市场建设，加强网络交易市场和快递行业监管。落实物流业发展中长期规划，加快随州综合物流园等重大物流项目建设，完善客货运站场布局。积极发展生活性 服务业，扶持壮大限上商贸企业，引导房地产业健康、规范发展，促进智能家电、文化创意等新型消费，进一步扩大医疗健康、家政服务、法律咨询等消费需求，促 进居民消费更加个性化、多样化。

（三）以更美环境彰显神韵随州新魅力。全面启动全国文明城市、卫生城市、环保模范城市、生态园林城市、双拥模范城新一轮“五城同创”，加快新型城镇化进程。

高标准推进城市建设。坚持以人为本，按照城乡统筹、产城融合的思路，启动编制城乡总体规划，做好重点区域、重点地段的控详规编制工作。做大做强政府融资平 台，积极探索资本市场融资，多渠道筹措资金73亿元，实施53项重大工程，加快新老城区互通互融。在新区建设上，依托路网、拓宽框架，建成东外环公路和城 南中环线，启动南外环公路建设，推进中国编钟音乐之都、城市公共活动综合体建设，进一步拓宽城市发展空间。在旧城改造上，全面推进功能配套、提档升级，加 快东护城河治理，启动交通大道明珠路至汉东路两侧环境综合整治，改造城市棚户区，新建美食文化街，维修老城区桥梁、排水设施，实施地下管网普查，加快智慧 城市建设，不断推进旧城焕新彩。

全方位提高城镇品位。加强城市规范化、科学化管理。加快完善“五城同创”必备要件，对标整改薄弱环节，不断放大创建边际效应。全面推行相对集中行政处罚权 管理，严格规划执法，坚决遏制违法违规建设行为。加快推进环卫作业、园林管护市场化，进一步规范小区物业管理。支持随县拓展东区、改造老城，支持广水市新 区建设和应广两城改造，支持曾都区城乡一体化发展，支持高新区打造生态产业新城。

多层次加强生态文明建设。着眼让随州地更绿，实施“绿满随州”行动，完成造林绿化28万亩，新建5个万亩特色林产业示范基地，新增城市绿地面积60万平方 米。着眼让随州水更清，大力推进城乡饮用水源地整治，全面封堵白云湖排污口，加大规模化畜禽养殖污染治理力度，加强城镇污水、垃圾处理设施建设和运行管 理。着眼让随州空气更清新，持续开展空气质量改善、城市噪音污染防治、黄标车和老旧车淘汰等专项行动，推进环保产业园建设，确保全面实现“十二五”减排目 标。着眼让随州乡村更美丽，推进农村生活垃圾治理全覆盖，加快美丽乡村示范片建设，继续抓好全国重点镇、省市级重点中心镇和特色镇建设，建成一批省级以上 生态村镇和宜居村庄。

（四）以更大力度开创改革开放新局面。坚持把深化改革作为今年政府工作的重大任务，继续实施开放先导战略，使市场的活力竞相迸发。

突出重点，坚定不移推进改革攻坚。深化政府机构改革，加快事业单位分类改革，组建市文化体育新闻出版广电局。深化农业农村改革，做好土地确权登记颁证工 作，构建与省统一联网、覆盖市县乡三级的农村综合产权交易体系，推行经营权抵押贷款，盘活土地、林地等资源，让农村的生产要素能够真正流动起来。深化企业 登记制度改革，全面推进注册登记联审联办“一表通”，启动新一轮国资国企改革。深化财税体制改革，大力推行零基预算，建立跨年度预算平衡机制，逐步实行政 府性债务分类预算管理，进一步扩大专项资金整合范围和规模。加快金融市场体系建设，积极引进股份制商业银行，新设1家村镇银行，深入开展政银企对接活动， 建立并用好企业续贷周转资金，力争新增贷款80亿元以上。力推泰晶科技上市和一批企业挂牌新三板、新四板。

内外并举，全面提升开放型经济水平。加强对外贸易形势动态分析，引导企业及时采取出口营销策略，稳定和拓展出口市场。完善扶持外贸出口政策措施，加快出口 基地建设，扩大专用汽车、食用菌、茶叶、通讯电子等出口产品规模，力争全年出口总额突破14亿美元。建好外派劳务服务中心和培训基地，加强技能培训和跟踪 服务，有序组织劳务输出。坚持引进来与走出去相结合，引导企业走出省门、国门，寻求更好地发展。

（五）以更实举措回应人民美好生活新期盼。

倾注深厚的感情做好民生工作，努力让全市人民更多、更公平地享受改革发展成果。

进一步完善社会保障体系。落实积极就业政策，深入推进全民创业，多渠道开发就业岗位，加强职业培训工作，重点解决高校毕业生、农村转移劳动力、城镇困难人 员、失业女工等群体创业就业问题，积极争创国家创业型城市。加大社会保险扩面征缴力度，适时调整参保人员待遇，完善被征地农民基本养老保险办法。继续推进 非公有制企业住房公积金建制扩面，进一步提高资金使用效益。健全完善城乡居民最低生活保障制度，抓好医疗救助和救灾救济工作。加快社会化养老服务体系建 设，全面拓展农村“两室联建”覆盖面。加快安居工程建设，不断改善低收入群众居住条件。

进一步统筹发展社会事业。继续抓好义务教育均衡发展，实施“全面改薄”工程，确保城东季梁学校、城南碧桂园学校交付使用，推进学前教育普惠发展；深化普通 高中课程改革，进一步提高教育质量；加快发展职业教育，大力推进产教融合、校企合作，服务经济社会发展。提升公共卫生服务能力，不断完善应急救助制度，扩 大基本药物实施范围，全面推进县级公立医院改革，加快市中心医院新院和中心血站建设，推进医疗、医药、医保“三医联动”，优化配置医疗卫生资源。抓好食品 药品安全示范创建工作，保障人民群众饮食用药安全。突出治理出生人口性别比，保持稳定适度的低生育水平。加快发展文化体育事业，加强历史文化名城保护，聚 集社会力量深度挖掘地域文化内涵;继续开展“送戏下乡”等文化惠民活动，启动广播电视户户通工程，广泛开展全民健身运动。充分展示随州改革发展成果，增强 “乡情乡愁”纽带，激发作为随州人的地域自豪感和归属感。

进一步提高社会治理水平。大力推进依法治市，启动创建省级法治城市，深入开展“六五”普法、“法律六进”等活动，不断提升全社会法治意识。优化社会治理体 系，加强社会治安防控体系建设，深化移动警务建设应用，推动警力下沉，严厉打击各类违法犯罪活动。继续抓好交通专项整治，促进城区交通秩序进一步好转。牢 固树立安全生产“红线”意识，严格落实党政同责、一岗双责、齐抓共管的要求，强化企业主体责任；深入开展隐患排查和重点领域整治，坚决遏制安全事故特别是 重特大事故发生。加强应急体系和应急能力建设，提升舆情处置水平。坚持需求导向，提高服务水平，打造“三民工程”升级版。大力推进城乡网格化和村务协理管 理模式，扩大基层民主自治范围。完善重大项目社会稳定风险评估等制度，健全完善社会矛盾纠纷大排查机制，加强人民调解、司法调解、行政调解“三调联动”， 确保跻身全省最平安城市行列。

进一步发展社会主义精神文明。大力培育和践行社会主义核心价值观，加强理想信念教育，积极引导人们重德厚德、尚德倡德。全面推进社会信用体系建设，加快打 造“信用随州”。积极开展双拥共建，密切军政军民关系。广泛开展文明社区、文明单位、文明村镇、十星级文明户等群众性精神文明创建活动，深入开展“做文明 有礼随州人”活动，着力整治不文明陋习，大力倡导文明交通、文明旅游、文明餐桌、文明上网和勤俭节约、孝老爱亲等时代风尚，全面凝聚正能量。

各位代表！通过听民声、察民情，今年市政府将重点办好以下民生实事：（1）扶持创业带动就业，新增就业2万人，增发创业就业小额担保贷款1亿元，扶持创业 1000人。（2）改善义务教育办学条件，投入1.2亿元，新改扩建教学及辅助用房2.2万平方米、生活用房2万平方米、运动场2.5万平方米，新建高新 区胜利小学。筹措资金4000万元，资助3万名贫困生。（3）启动市图书馆新馆、科技馆、妇儿中心、老年大学、残疾人康复中心建设，建成城乡健身设施60 处、农村（社区）文化广场50个。（4）推进农村“两室联建、医养融合”；加快建设居家养老信息服务平台，打造主城区“十分钟为老服务圈”。（5）实施保 障性安居工程，建设500套公共租赁房和3000户城市棚户区改造住房。改造主城区50座公共旱厕。（6）创新、完善立体化社会治安防控体系，加快物防技 防设施建设，建立反恐防暴快速处置和网格化巡逻防控体系。新建主城区平安车场10个、平安车棚20个、平安停车点100个。（7）加强城区饮用水源地保护 与综合治理，关闭王福窑取水口、调整为备用水源地，全部清理拆除先觉庙、封江水库筑坝拦汊、拦网及网箱等养殖设施；开展乡镇集中式饮用水源地规范化建设试 点工作。再解决20万农村人口的安全饮水问题。（8）继续开展“洁美家园大行动”和农村生活垃圾专项治理。实施精准扶贫，确保整村推进20个、结对帮扶6 万户，减贫脱贫2万人以上。（9）加快便民市场建设，治理规范城区农贸市场，完成黄垅市场整体搬迁，兴建随州农副产品物流园，新配建小区便民蔬菜店10处 以上。（10）实施主城区畅通工程，疏通部分瓶颈道路，续建季梁大道，延伸解放东路，改造水一桥，搬迁中心客运站，完善配套一批停车场，建设一批公交枢 纽站、首末站，增开公交线路，规范交通标识标牌，努力缓解主城区交通拥堵问题。

四、切实加强政府自身建设

面对新形势、新常态、新要求，必须更好地发挥政府的作用，进一步抓好政府自身建设。

以依法行政为重点，致力建设法治政府。把依宪依法行政作为政府工作的基本遵循，切实做到法定职责必须为、法无授权不可为。深入贯彻落实省委关于《全面推进 法治湖北建设的意见》，着力提升各级政府及其部门法治思维和依法办事的能力，全面提高公务员法律素养。依法明确各级政府事权，强化行政首长推进依法行政第 一责任，将其出庭应诉情况纳入考核范围。坚持科学民主决策，严格遵守重大行政决策法定程序，建立健全重大决策合法性审查、终身责任追究及倒查机制，加强规 范公正文明执法。完善监督制度体系，自觉接受人大及其常委会的法律监督，主动接受政协民主监督，认真办好建议提案。加强法制、司法机构建设，继续做好法律 援助、司法救助、行政复议、仲裁等工作，全面推进建立法律顾问制度。做好政务公开工作，切实保障人民群众的知情权、参与权和监督权。着力解决信访突出问 题，畅通渠道，规范程序，逐步实行网上受理，推行访诉分离新机制，依法严厉打击“三堵五闹”行为。

以转变职能为核心，致力建设效能政府。进一步转变政府职能，探索建立“四联单”运行机制，全面推进行政提速。加快晒出“权力清单”，精简权力事项，定期清 理、实时公开，并划定每个单位、每个岗位的职权边界。加快实施行政审批“负面清单”制度，明确禁止和限制投资领域，建立行政审批事项统一目录和联合审批大 平台，推行“一口受理、联合审批、综合执法”服务模式，加快审批提速，规范审批收费。加快建立“责任清单”，完善岗位目标考核体系，构建执行落实链条，整 治为官不为和行政不作为、慢作为、乱作为，强化检查督办、跟踪问效，以“钉钉子”的精神抓好落实。加快优化“程序清单”，巩固扩大“三集中三到位”改革成 果，全面落实“三短一简一俭”，切实提高办事效率。

以解放思想为先导，致力建设创新政府。坚持与时俱进，大兴学习调研之风，上好“市场大学”、学好“市场专业”，吃透政策，更新思维，不断提高新常态下驾驭 复杂局面的能力。增强先行先试的胆略，加强特色发展战略、产业、项目等方面的科学谋划，把握规律、大胆探索、差异竞争，争创全省乃至全国改革试点示范，力 求在更多领域创造鲜活的“随州经验”。紧盯“保五争三”的目标，创新工作方式，千方百计保持较快的经济增长速度，提高发展的质量效益，努力推动各项工作在 全省争先进位、争创一流。

以服务群众为根本，致力建设为民政府。持续巩固扩大教育实践活动成果，坚持把群众的呼声、期待和诉求作为施政理政的出发点和落脚点，把服务企业、基层和群 众的各项工作做深、做细、做扎实。突出问题导向，建立侵害群众利益行为专项治理长效机制，重拳整治“三乱”行为，着力优化企业发展环境。全面落实惠民政 策，打通“最先一公里”和“最后一公里”，强化各项惠民举措，尤其要高度关注、切实保障低收入群众的基本生产生活，及时帮助他们解决实际困难。

以制度建设为保障，致力建设廉洁政府。坚持从严治政，严明党的政治纪律和政治规矩，坚决执行中央、省委和市委各项禁令，纠正“过头论”、“刮风论”等模糊 认识。健全完善作风建设各项制度，持续加大“四风”问题整改力度，强化电视问政、政风行风评议、明察暗访等有效措施，绵绵用力、久久为功，不断把作风建设 引向深入。大力推进清廉随州建设，认真执行党风廉政建设责任制，切实落实主体责任，严格责任追究，扎实推进廉政风险防控体系建设。严控“三公”经费支出， 坚决反对奢侈浪费，以勤政廉政、务实有为的实际成果取信于民。

各位代表！随州正处在改革攻坚、转型发展的关键时期，时代赋予重任，实干铸造辉煌。让我们在市委的正确领导下，汇聚全市人民的智慧和力量，主动适应新常态，满怀激情续新篇，为成就“圣地车都”美好梦想、加快全面建成小康社会而努力奋斗！
